# Peroxisome Proliferator-Activated Receptor**α** Agonists Differentially Regulate Inhibitor of DNA Binding Expression in Rodents and Human Cells

**DOI:** 10.1155/2012/483536

**Published:** 2012-06-04

**Authors:** María del Carmen González, J. Christopher Corton, Nuria Acero, Dolores Muñoz-Mingarro, Yolanda Quirós, Juan José Álvarez-Millán, Emilio Herrera, Carlos Bocos

**Affiliations:** ^1^Departmento de Biología, Facultades de Farmacia y Medicina, Universidad San Pablo-CEU, Urbanización Montepríncipe, Boadilla del Monte, 28668 Madrid, Spain; ^2^Integrated Systems Toxicology Division, National Health and Environmental Effects Research Lab, US Environmental Protection Agency, 109 T.W. Alexander Drive, Research Triangle Park, NC 27711, USA; ^3^Consulting Químico Sanitario (CQS) Laboratory, 28020 Madrid, Spain

## Abstract

Inhibitor of DNA binding (Id2) is a helix-loop-helix (HLH) transcription factor that participates in cell differentiation and proliferation. Id2 has been linked to the development of cardiovascular diseases since thiazolidinediones, antidiabetic agents and peroxisome proliferator-activated receptor (PPAR) gamma agonists, have been reported to diminish Id2 expression in human cells. We hypothesized that PPAR**α** activators may also alter Id2 expression. Fenofibrate diminished hepatic Id2 expression in both late pregnant and unmated rats. In 24 hour fasted rats, Id2 expression was decreased under conditions known to activate PPAR**α**. In order to determine whether the fibrate effects were mediated by PPAR**α**, wild-type mice and PPAR**α**-null mice were treated with Wy-14,643 (WY). WY reduced Id2 expression in wild-type mice without an effect in PPAR**α**-null mice. In contrast, fenofibrate induced Id2 expression after 24 hours of treatment in human hepatocarcinoma cells (HepG2). MK-886, a PPAR**α** antagonist, did not block fenofibrate-induced activation of Id2 expression, suggesting a PPAR**α**-independent effect was involved. These findings confirm that Id2 is a gene responsive to PPAR**α** agonists. Like other genes (apolipoprotein A-I, apolipoprotein A-V), the opposite directional transcriptional effect in rodents and a human cell line further emphasizes that PPAR**α** agonists have different effects in rodents and humans.

## 1. Introduction

Fibrates have been effectively used to reduce plasma triglyceride levels under conditions of hypertriglyceridemia [1]. The molecular basis for the action of fibrates on lipid metabolism involves the activation of transcription factors, known as peroxisome proliferator-activated receptors (PPARs), principally the PPAR*α* subtype expressed in liver ([2], for a review). Fibrates decrease the gene expression of apolipoprotein C-III, and increase the expression of fatty acid-catabolizing enzymes like acyl-coenzyme A oxidase [3] and 17*β*-hydroxysteroid dehydrogenase (17*β*-HSD) type IV [4] in rodent liver. Fibrates also display other effects, not directly related to the lowering of plasma lipids, including the modulation of immune and inflammatory responses. Thus, these drugs downregulate acute-phase protein expression, such as fibrinogen, C-reactive protein, and *α*2-macroglobulin [5–7].

PPARs also play an important role in glucose homeostasis. PPAR*α* agonists, by upregulating fatty acid oxidation and ketone body production, are able to spare glucose. Several studies have indicated a beneficial effect of PPAR*α* activation on insulin sensitivity [8, 9]. Thus, hyperinsulinemia and hyperglycemia observed in mice subjected to a high-fat diet or in genetic insulin-resistant rodents [8] were sharply reduced by treatment with fibrates. The antidiabetic thiazolidinediones (TZD) drugs which are ligands of the PPAR*γ* subtype, are prescribed for regulating glucose metabolism because they lower blood glucose by enhancing peripheral insulin sensitivity [10].

It has been shown that the levels of Id2, a member of the helix-loop-helix (HLH) transcriptional repressor protein family which includes Id1-4 [11, 12], are reduced in aortic smooth muscle cells by treatment with TZD suggesting that Id2 might play a role in their antidiabetic effects [10]. Furthermore, since glucose increases Id2 protein levels, Id2 could contribute to changes in cellular function that occur in insulin-resistant and diabetic states [13]. Interestingly, Id2 is upregulated in muscle, fat, and liver of obese ob/ob mice [14]. Park et al. [15] have demonstrated that Id2 is a transcriptional modifier of PPAR*γ* expression and adipogenesis and found that Id2 expression is elevated in adipose tissues of diet-induced obese mice and humans leading to the hypothesis of a role for Id2 in obesity and insulin resistance. Furthermore, Id2 nullizygous mice show altered expression of genes involved in lipid metabolism which could be related to reduced lipid storage in liver and white adipose tissue [16]. These authors also found that genes involved in glucose homeostasis exhibited altered expression in Id2-null mice.

Id proteins participate in development, cell cycle control, differentiation, and tumorigenesis [17]. Id2 protein heterodimerizes with E proteins, a subset of basic HLH (bHLH) transcription factors [18] and sterol regulatory element-binding protein-1c (SREBP)-1c [19], but because Id2 lacks a DNA binding domain, Id2 acts as a dominant negative regulator of these transcription factors [11]. Additionally, Id2 is able to regulate the function of HLH transcription factors indirectly by sequestering E proteins [18].

Changes in lipid metabolism and insulin resistance during late pregnancy are comparable to that normally seen in type 2 diabetic patients, in which the use of fibrates is recommended [20]. For that reason, late pregnancy has been previously used by our group [2, 21–24] and other authors [25–28] to study the effect of PPAR agonists. We have used these experimental settings to discover new PPAR*α* target genes in rodents [7]. Thus, we have used late-gestation rats to study the effect of fibrates in hepatic Id2 mRNA expression. In addition, since free fatty acids (FFA) are known to act as PPAR*α* activators and fasting increases circulating FFA [29–31], the role of FFA on the Id2 mRNA expression was investigated in fasted rats. Furthermore, in order to determine whether the effect of fibrates on Id2 gene expression is mediated by PPAR*α*, wild-type and PPAR*α*-null mice were used. Finally, in order to study whether the effect of fibrates on Id2 gene expression is species-specific, the human hepatocarcinoma cell line (HepG2) was used as a model system.

## 2. Materials and Methods

### 2.1. Animals, Drug Administration, and Collection of the Samples

**Study I**

Female Sprague-Dawley rats weighing 180–210 g were fed *ad libitum* standard rat chow (B&K Universal, Barcelona, Spain) and housed under controlled light and temperature conditions (12 h light-dark cycle; 22 ± 1°C). The experimental protocol was approved by the Animal Research Committee of the University San Pablo-CEU, Madrid, Spain. Half the animals were mated, and day 0 of pregnancy was determined by the appearance of spermatozoids in vaginal smears, whereas the remaining half were kept virgin. From day 16 of gestation, rats were given by oral gavage two daily doses of 0, 100 or 200 mg of fenofibrate (Sigma-Aldrich, St Louis, MO, USA)/kg of body weight, one at 8.00 h and the other at 18.00 h, suspended in 2% Tween-80 or Tween-80 alone. On the morning of the 20th day of pregnancy (after 4 days of treatment), corresponding to 14 h after receiving the last treatment, rats were decapitated and blood collected using tubes containing Na_2_-EDTA. Liver was immediately removed, placed in liquid nitrogen and kept at −80°C until analysis. Virgin rats received the same treatment and were studied in parallel. There were 5-6 animals per group.

**Study II**

Female Sprague-Dawley rats weighing 180–210 g were mated, and half the animals were subjected to fasting for 24 h at day 19 of pregnancy. At day 20 of pregnancy, blood and liver were collected as before. Plasma aliquots were kept at −20°C until processing for the analysis of FFA by enzymatic commercial kit (Wako Chemicals GmbH, Neuss, Germany).

**Study III**

Male SV129 wild-type mice were purchased from Taconic (Germantown, NY, USA), and male SV129 PPAR*α*-null mice [32] were a kind gift from Frank Gonzalez (National Cancer Institute, Bethesda, MD, USA). Control and treated mice (*n* = 2–5) were provided NIH-07 rodent chow (Ziegler Brothers, Gardner, PA, USA) and water *ad libitum*. This study was conducted under federal guidelines for the use and care of laboratory animals and was approved by the Chemical Industry Institute of Toxicology Institutional Animal Care and Use Committee (NC, USA). Lighting was on a 12 hr light/dark cycle. Mice were fed diet supplemented with either Wy-14,643 (WY) (ChemSyn Science Laboratories, Lenexa, KS, USA) (0.1%) or di-(2-ethylhexyl)phthalate (DEHP) (Aldrich Chemical, Milwaukee, WI, USA) (0.6%), or a control diet for 3 weeks. WY and DEHP were selected because of their different structural properties and uses. DEHP is considered a weak PPAR activator compared to WY. At the designated time after treatment, animals were anesthetized by pentobarbital injection and killed by exsanguination. Livers were removed, rinsed with isotonic saline, snap-frozen in liquid nitrogen, and stored at −80°C until analysis.

**Study IV**

Human hepatocarcinoma cells (HepG2) were obtained from American Type Culture Collection (HB-8065) (Manassas, VA, USA) and cultured in EMEM media, supplemented with 1% glutamine, 1% nonessential amino acids, 3% antibiotics (100 U/mL penicillin and 100 *μ*L/mL streptomycin), and 10% fetal bovine serum. All cells were grown in a 5% CO_2_-humidified atmosphere at 37°C. After confluence, cells were cultured in serum-free medium (with 0.1% BSA) for 24 hours and different concentrations of fenofibrate (0, 10, 50, and 100 *μ*M) in DMSO were added. After different times of incubation (2, 6, and 24 hours), media was collected and cells were washed with ice-cold PBS and removed with a cell scraper. After centrifugation, cell pellets were frozen and used for RNA extraction. In some cases, cells were preincubated for 30 minutes [33] with the PPAR*α* antagonist MK-886 (Enzo Life Sciences Inc., Farmingdale, NY, USA) (10 *μ*M) dissolved into DMSO. DMSO concentration in culture medium did not exceed 0.1%. An additional experiment was carried out in the same conditions as described above but the cells were instead cultured in serum-free medium for 36 hours, and then treated with the drugs.

### 2.2. Total RNA Preparation and Analysis

**Studies I and II**

Rat total hepatic RNA was isolated by a modification of the guanidium isothiocyanate method using Ultraspec RNA according to the manufacturer's instructions (Biotecx Labs, Houston, USA). Total RNA concentration was determined by absorbance measurement at 260 nm. The 260/280 absorption ratio of all samples was between 1.8 and 2.0. Total RNA-genomic DNA-free samples were used to analyse the expression of Id2 gene and glyceraldehyde-3-phosphate dehydrogenase (GAPDH) as endogenous control, by semiquantitative RT-PCR, according to previously described protocols [7, 24]. Briefly, total RNA (2.5 *μ*g) was digested with 5 U RNase free-DNaseI (Roche, USA) for 20 minutes at 37°C to remove traces of genomic DNA. The DNase was inactivated at 64°C for 10 minutes and cDNA was synthesized from total RNA by oligo(dT)-primed reverse transcription with Superscript II (Invitrogen, Life Technologies Ltd., Paisley, UK), according to the manufacturer's instructions. PCRs were performed in a 25 *μ*L reaction mix containing 20 pmol of both forward and reverse primer, 10 mmol/L of each deoxyribonucleoside triphosphate, appropriate dilutions of the cDNA stock, 2.5 *μ*L of PCR 10X buffer, and Accu Taq-polymerase (Sigma-Aldrich, St Louis, MO, USA). The sense and antisense primer sequences were 5′-GAAAAACAGCCTGTCGGACCA-3′ and 5′-CCAGGGCGATCTGCAGGT-3′ for Id2 (205 bp product); and 5′-ACCACAGTCCATGCCATCAC-3′ and 5′- TCCACCACCCTGTTGCTGT-3′ for GAPDH (450 bp product) [34, 35].

All reactions were performed in a PTC-100 Thermocycler (MJ Research, USA) in which samples underwent a 3 min initial denaturing step, followed by 35 cycles of 45 s to 1 min at 94°C, 45 s at the annealing temperature of 65°C for Id2 and 57°C for GAPDH, and a primer extension step at 72°C for 45 s to 1 min. The final extension step was 10 min at 72°C. The PCR products were analysed by agarose gel electrophoresis and DNA was visualized by ethidium bromide staining and using a UV-light box. Band intensity was determined by quantitative scanning densitometry (GS-700 Imaging Densitometer, BioRad, Hercules, CA, USA). To determine the linear range of the PCR, dilutions of the cDNA preparations were previously used for each gene and experimental group of rats. Results were normalized to the control gene (GAPDH).

**Study III**

Total RNA was isolated from mouse livers by a modification of the guanidinium isothiocyanate method using RNAzol according to manufacturer's instructions (Tel-Test, Friendswood, TX, USA). Twenty *μ*g of denatured total RNA was separated on 1.2% agarose gels and transferred to nylon membranes in 20x SSC. The DNA probes for Northern blot analysis were labeled with [*α*-^32^P]dCTP using the random primer DNA labelling kit provided by Amersham. Probes used were a rat L-bifunctional enzyme (Ehhadh) cDNA fragment, the complete cDNA of rat 17*β*-HSD type IV [4] and the PCR products generated as indicated above in the studies I and II, and using rat cDNA as a template. The probes were sequenced (ABI PRISM 377 Perkin Elmer DNA sequencer), and the sequences obtained were compared to Gene Bank sequences to confirm the accuracy of the probes used. Blots were prehybridized at 42°C for 2 h and hybridized overnight at the same temperature. Washing conditions were 0.1x SSC, 0.1% SDS at 53°C for 15 min three times, and membranes were exposed to appropriate screens (Imaging Screen K, BioRad) at 4°C from 1 h to three days and the images analyzed (Personal Molecular Imager FX, BioRad). Filters were stripped of label at 75–80°C for 1 h with 0.1x SSC, 0.5% SDS, 0.1% tetrasodium pyrophosphate and then rehybridized.

**Study IV**

Total RNA was isolated from HepG2 cell pellets by QIAcube automated protocol using spin-column kit (RNeasy Mini Kit, QIAGEN, Hilden, Germany). Total RNA concentration was determined by absorbance measurement at 260 nm. The 260/280 absorption ratio of all samples was between 1.8 and 2.0. Total RNA-genomic DNA-free samples were used to analyse the expression of Id2 and *β*-actin as a control, by reverse transcription and quantitative real time PCR (qPCR) assays, according to the following protocol: cDNA was synthesized from 1 *μ*g total RNA by Transcriptor high fidelity cDNA synthesis kit (Roche, Mannheim, Germany), according to the manufacturer's instructions. qPCRs were performed in a 20 *μ*L reaction mix containing 20 pmol of both forward and reverse primer, SYBR Premix Ex Taq (Takara Bio Inc., Tokyo, Japan) and cDNA. Sense and antisense primer sequences were 5′-GAAAGCCTTCAGTCCCGTGAGGTCCGTT-3′ and 5′-CTGGTGATGCAGGCTGACAATAGTGGGATG-3′ for Id2 (271 bp) (Atlas RT-PCR Primer Sequences (Clontech, CA, USA); 5′-CCTGGCACCCAGCACAAT-3′ and 5′-GGGCCGGACTCGTCATAC-3′ for *β*-actin (145 bp) [36]. Samples were analyzed in duplicate. All reactions were performed in a LightCycler 5.0 (Roche). Optimal qPCR efficiency and linearity were previously confirmed for each target. Results for the expression of Id2 mRNA were expressed relative to the control gene (*β*-actin).

### 2.3. Statistical Analysis

Results were expressed as means ± S.E. Treatment effects were analyzed by one-way analysis of variance (ANOVA). When treatment effects were significantly different (*P* < 0.05), means were tested by Tukey multiple range test. For nonparametric data, the Mann-Whitney *U* test was used with differences between the two groups analyzed by Student *t*-test.

## 3. Results

### 3.1. Effect of Fenofibrate on Id2 Expression in Pregnant and Virgin Rats

**Figure 1:**
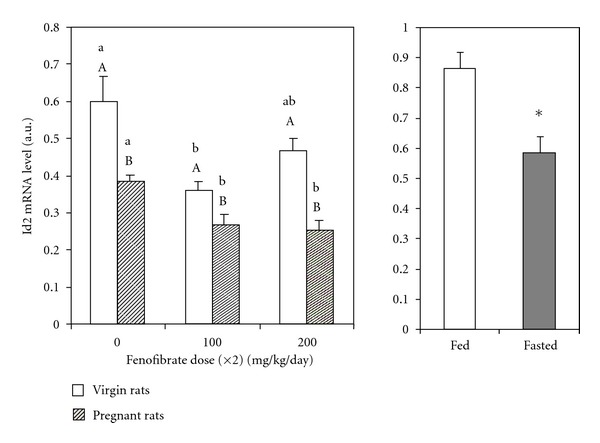
Fenofibrate downregulates Id2 gene expression in rats. Left panel: relative amount of mRNA of liver Id2, after 4-day treatment with or without fenofibrate in virgin and pregnant rats, measured by semiquantitative RT-PCR. Values were normalized against glyceraldehyde-3-phosphate dehydrogenase (GAPDH) expression and were represented using arbitrary units. Capital letters correspond to the statistical comparisons between pregnant and virgin rats receiving the same treatment. Small letters correspond to the statistical comparisons between rats receiving different drug doses. Values not sharing a common letter are significantly different at *P* < 0.05. Each value represents the mean ± standard error of five animals. Right panel: starvation downregulates Id2 gene expression. Relative amount of mRNA of liver Id2 from pregnant rats fed with standard pellet or fasted 24 h, measured by semiquantitative RT-PCR. Values were normalized against GAPDH expression and were represented using arbitrary units. Asterisk represents significantly different at *P* < 0.05.

As shown in Figure 1, hepatic Id2 mRNA levels were higher in virgin than in pregnant rats in the absence of treatment. In nonpregnant rats, hepatic Id2 mRNA levels were decreased by treatment with fenofibrate, although the effect at higher dose was not significant. PPAR*α* agonist treatment for 4 days also decreased Id2 mRNA levels in pregnant rats (Figure 1) independently of the dose used, indicating that the lower dose was sufficient to reduce the expression of the Id2 gene. These results validate those previously found by our group when Id2 levels were evaluated using the same samples by macroarray technology (Atlas Nylon Arrays, Clontech, BD Biosciences, Palo Alto, CA, USA) (unpublished results).

### 3.2. Effect of Fasting on Id2 Expression

It is well known that several types of fatty acids are PPAR*α* activators [29, 30]. The uptake of fatty acids into the liver as a result of their mobilization from adipose tissue after fasting would result in PPAR*α* activation and changes in the expression of its target genes [31]. Plasma FFA levels in fed rats were 360.84 ± 22.15 *μ*M, significantly different (*P* < 0.05) from those levels found in 24 h fasted rats: 1,503.90 ± 157.81 *μ*M. As shown in Figure 1, 24 h fasting produced a significant decrease in hepatic expression of Id2 in comparison to the rats fed *ad libitum* correlating to the increase in circulating fatty acids.

### 3.3. Requirement for PPAR*α* in Fibrate Regulation of Id2 Expression

**Figure 2:**
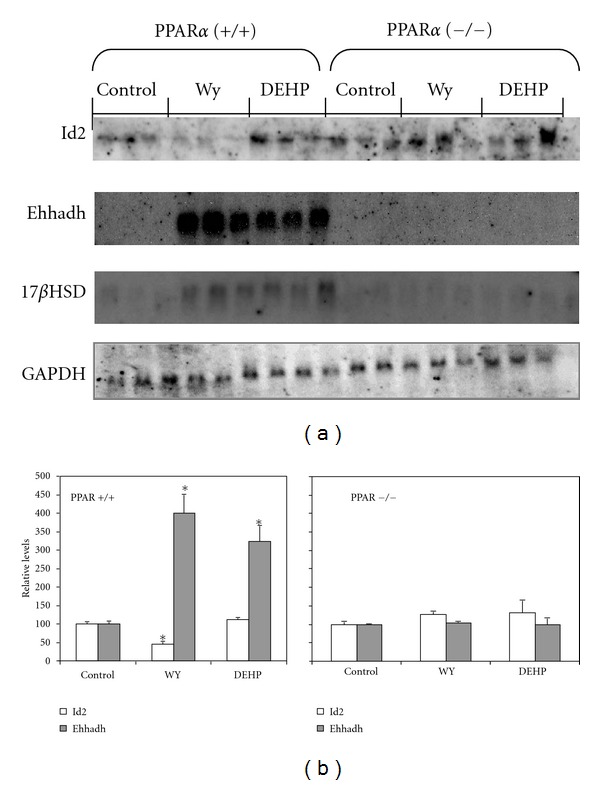
Downregulation of Id2 gene expression by WY is dependent on PPAR*α*. Wild-type SV129 mice (+/+) or SV129 mice that lack PPAR*α* (−/−) were fed a control diet (*Control*) or a diet containing WY (0.1%) or DEHP (0.6%) for 3 weeks. Total RNA isolated from liver was separated by 1.2% agarose, transferred to nylon, and analysed by northern blot using probes for Id2, Ehhadh, and 17*β*HSD, and GAPDH as a control. Northern autoradiograms (a) were densitometrically scanned and expression normalized to that of GAPDH (b). Each value represents the mean ± standard error of three animals. *significantly different from control (*P* < 0.05).

Because PPAR*α* has been shown to mediate several fibrate-inducible responses in the liver, we examined the dependence of fibrate-induced decreases in Id2 gene regulation on PPAR*α* expression. Wild-type mice and PPAR*α*-null mice [32] were fed a control diet or the same diet supplemented with either WY (0.1%) or DEHP (0.6%) for 3 weeks. As shown in Figure 2, when wild-type mice were fed WY there was a significant decrease in the liver expression of Id2 mRNA, whereas treatment with DEHP, a weaker PPAR*α* activator, did not change the levels of Id2 mRNA. Treatment of PPAR*α*-null mice with WY or DEHP resulted in no change in Id2 gene expression (Figure 2).

As a positive control of PPAR*α* agonist regulation of gene expression, we also examined the levels of L-bifunctional enzyme (Ehhadh) mRNA. As expected [37, 38], Ehhadh mRNA expression was significantly enhanced by WY or DEHP treatments in wild-type mice but not in PPAR*α*-null mice (Figure 2). A similar effect was found for 17*β*-HSD type IV gene expression (Figure 2), in accordance with our previously published results [4]. As a negative control, GAPDH mRNA levels remained constant under all conditions (Figure 2).

### 3.4. Effect of Fenofibrate on Id2 Expression in Human Cultured Cells

**Figure 3:**
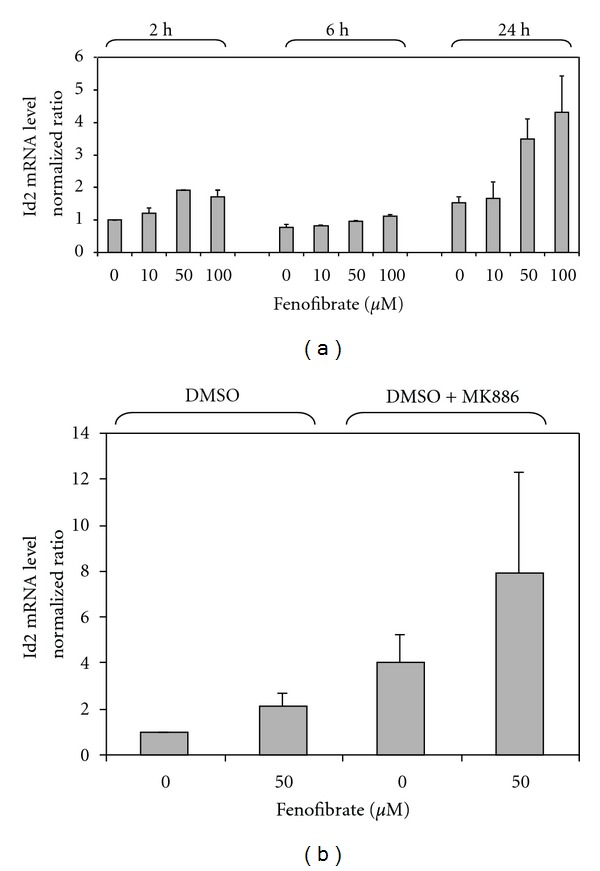
Fenofibrate upregulates Id2 gene expression in HepG2 cells. Panel (a) human hepatocarcinoma cells treated with different concentrations of fenofibrate (0, 10, 50, or 100 *μ*M) for 2, 6, or 24 hours. Relative Id2 mRNA levels were measured by real-time PCR, normalized to *β*-actin levels and expressed in relative units to control. Values for Id2 mRNA are expressed as mean ± SD (*n* = 3). Panel (b) HepG2 cells were preincubated with the PPAR*α* antagonist MK-886 (10 *μ*M) where indicated and treated with different concentrations of fenofibrate (0 or 50 *μ*M) for 24 hours.

Since fibrates depressed Id2 hepatic expression in rodents in a PPAR*α* mediated manner, we also determined if Id2 mRNA expression exhibits a similar behaviour in human cells. Unexpectedly, fenofibrate at 50 and 100 *μ*M increased Id2 mRNA expression after 24 hours of treatment (Figure 3(a)). A previous report has shown that glucose could induce Id2 expression in cultured cells [13]. The EMEM media used here contained 5 mM glucose, therefore, we repeated the experiment in the presence of 20 mM glucose. The results observed in the presence of additional glucose were similar to those described in Figure 3(a) (data not shown).

To determine if the activation of Id2 expression is mediated by PPAR*α* in HepG2 cells, the cells were preincubated with the PPAR*α* antagonist MK-886 [39]. As shown in Figure 3(b), the effect of fenofibrate was not blocked by preincubation with MK-886, indicating that the induction was independent of PPAR*α*. Instead, the PPAR*α* antagonist showed an additive effect with fenofibrate on Id2 mRNA expression (Figure 3(b)). Glutathione S-transferase pi 1 (Gstp1), which expression has been recently shown to be modified by PPAR*α* activators [40], was also measured. Fenofibrate increased Gstp1 expression (1.7-fold induction versus control without drug), whereas MK-886 abolished the effect of fenofibrate on HepG2 cells.

A recent report showed that Id2 expression could be influenced by circadian rhythm [16], and it has been previously established that serum is able to induce circadian gene expression [41, 42]. Therefore, an additional experiment was carried out with MK886 and fenofibrate, in which the cells were cultured in serum-free medium for 36 hours instead of 24 hours. Similar results to those observed in Figure 3(b) were obtained when the serum was substituted with BSA, 36 hours before the treatment with the drugs (data not shown).

## 4. Discussion

In this study, we found that fenofibrate treatment repressed liver Id2 mRNA expression both in pregnant and virgin rats. These findings agree with those of Yamazaki et al. [43] using cDNA microarrays from mice after 2 or 3 days oral administration of fenofibrate (100 mg/kg) or WY (30 mg/kg), and are consistent with those reported by Wong and Gill [44] after 1.0% DEHP in the diet for 13 weeks as studied by microarrays.

Glucocorticoids, whose circulating levels are augmented during pregnancy [45], have been described as repressors of Id2 expression in cells [46]. In agreement with this, basal levels of Id2 mRNA in pregnant rats were lower than in unmated rats, the difference being also observed after fenofibrate treatment. These findings emphasize the downregulatory effect of PPAR*α* agonists, independent of whether Id2 mRNA levels are low, as in gestation, or elevated as in nonpregnant rats.

Fasting produces mobilization of fatty acids from adipose tissue. Fatty acids are natural activators of PPAR*α* [29, 31, 47]. Therefore, the arrival of fatty acids in the liver as result of starvation, led to a significant decrease in hepatic expression of Id2. This finding reinforces the idea that PPAR*α* activation produces a decrease in Id2 mRNA expression in liver.

Id2-null mice exhibit a decrease in adipose tissue and liver fat deposition compared to wild-type mice [16]. Consistent with that, Id2-overexpressing adipocytes show increased capacity for morphological differentiation and lipid accumulation [15]. In contrast, we found that PPAR*α* activators decrease Id2 gene expression under the same conditions as we had previously reported an accumulation of lipids in the liver. Thus, hepatic triglyceride content in fasted rats was higher in comparison to fed condition [48], and it was also augmented in nonpregnant fenofibrate treated rats (in comparison to nontreated unmated rats) [24]. Nevertheless, although Id2 mRNA expression was also modified by fenofibrate in late gestation, hepatic triglyceride content was not affected by the drug in pregnant rats [24].

Since it has been reported that Id2 inhibits lipogenesis by interfering with the transcriptional activity of SREBP1c at the fatty acid synthase (FAS) promoter [19], our results might reflect an increased lipogenesis along with an Id2 repression. However, the hepatic expression of FAS was not significantly changed by fenofibrate in virgin or in pregnant rats [24], and 24 h fasting instead decreased FAS expression in pregnant rats (unpublished results).

We found a decrease in the expression of Id2 in mice treated with WY-14,643, yet this effect was not seen with DEHP, indicating that the effect depends on PPAR*α* activator potency. In the case of mice that lacked a functional form of PPAR*α*, differences were not observed after the different treatments, suggesting that it was an effect mediated by PPAR*α*. This finding is in agreement with that one recently described by el Azzouzi et al. [49] in murine cardiomyocyte cells using cDNA microarrays. One of gene listed by these authors to be specifically downregulated by WY was Id2. However, neither GW-5015160 (a PPAR*β*/*δ* agonist) nor rosiglitazone (a PPAR*γ* agonist) produced any change. Thus, it can be assumed that fibrates affect Id2 gene expression through PPAR*α*.

The decrease found in the expression of Id2 in mice by the potent PPAR*α* activator WY agrees with the reduction observed in rats treated with fenofibrate. Since, it appears that both STAT3 and C/EBP*β* are involved in regulation of Id2 [50, 51], and it is known that PPAR*α* activation interferes with signalling pathways dependent on C/EBP and STAT [52], we hypothesize that PPAR*α* may negatively regulate Id2 through inhibition of STAT3 or C/EBP.

In contrast to rodents, the negative effect of fibrates on Id2 gene expression was not observed in human cells. Fenofibrate enhanced Id2 mRNA levels in these cells after a 24 h incubation. Moreover, since Grønning et al. [13] have shown in murine macrophages that glucose induces increases in protein levels of this transcriptional repressor, we studied the effect of fibrates both at low glucose and high glucose and found a similar fenofibrate-inducing effect on Id2 mRNA expression. The effect of fenofibrate was observed after 24-h incubation, suggesting that regulation of Id2 expression by PPAR might occur by an indirect mechanism [53]. Nevertheless, several PPAR*α* target genes [54] were also induced after 24-hour administration of fibrates but not earlier. Therefore, we studied the effect of MK-886, an antagonist of PPAR*α* and found that fenofibrate-induced increases in Id2 mRNA expression were not abolished by preincubation with MK-886, confirming that the effect was not mediated by PPAR*α*. In accordance with this finding, TZD, which also modulate Id2 mRNA levels in cultured human cells, use a PPAR*γ*-independent mechanism [10]. Fenofibrate and MK-886 functioned synergistically to stimulate Id2 expression. The MK-886-induced increase in Id2 mRNA expression could be caused by two mechanisms: (i) specific inactivation of PPAR*α*; (ii) other pathways, such as inhibition of leukotriene biosynthesis [39]. Therefore, it is assumed that fenofibrate-induced increases in Id2 expression occur by a PPAR*α*-independent mechanism. How fenofibrate increases Id2 expression in HepG2 cells remains elusive.

Finally, Id proteins are HLH transcription factors that participate in development, cell cycle control, differentiation, and tumorigenesis [17]. However, the role of Id2 protein in the mechanism of action of fibrates has not been elucidated. Altogether, these findings confirm that Id2 gene expression is responsive to PPAR*α* activators (fibrates and possibly fatty acids). However, as reported for other genes (apoA-I, apoA-V) ([2] and references therein), the effects are opposite in rodents versus humans. Since peroxisome proliferators function as hepatocarcinogenic agents in rodents, but not in humans [55], and considering the role of Id2 protein in cell proliferation and cancer [17], we speculate that the differential response to fibrate exposure might be related to the differences in liver tumorigenesis between species.

## Abbreviations

PPAR: Peroxisome proliferator-activated receptor
GAPDH: Glyceraldehyde-3-phosphate dehydrogenase
Id2: Inhibitor of DNA binding 2
DEHP: Di-(2-ethylhexyl)phthalate
WY: WY-14,643
RT-PCR: Reverse transcriptase polymerase chain reaction
EMEM: Eagle's minimal essential medium
A.U.: Arbitrary units.
